# Pediatric faculty and residents’ perspectives on In-Training Evaluation Reports (ITERs)

**Published:** 2015-12-11

**Authors:** Rikin Patel, Anne Drover, Roger Chafe

**Affiliations:** 1 Division of Pediatrics, Memorial University of Newfoundland; 2 Faculty of Medicine, Memorial University of Newfoundland

## Abstract

**Background:**

In-training evaluation reports (ITERs) are used by over 90% of postgraduate medical training programs in Canada for resident assessment. Our study examined the perspectives of faculty and residents in one pediatric program as a means to improve the ITER as an evaluation tool.

**Method:**

Two separate focus groups were conducted, one with eight pediatric residents and one with nine clinical faculty within the pediatrics program of Memorial University’s Faculty of Medicine to discuss their perceptions of, and suggestions for improving, the use of ITERs.

**Results:**

Residents and faculty shared many similar suggestions for improving the ITER as an evaluation tool. Both the faculty and residents emphasized the importance of written feedback, contextualizing the evaluation and timely follow-up. The biggest challenge appears to be the discrepancy in the quality of feedback sought by the residents and the faculty members’ ability to do so in a time effective manner. Others concerns related to the need for better engagement in setting rotation objectives and more direct observation by the faculty member completing the ITER.

**Conclusions:**

The ITER is a useful tool in resident evaluations, but a number of issues relating to its actual use could improve the quality of feedback which residents receive.

## Introduction

Post-graduate medical residents require regular and constructive assessment during their training to guide their professional development and ensure that they meet the standards of competency required for medical professionals. An effective assessment system is crucial for ensuring that appropriate development is taking place, that assessment is supporting learning, and that standards of clinical competency are being met. The in-training evaluation report (ITER), based on the observation and documentation of the performance of learners in real clinical settings, is the cornerstone of the assessment system in most North American medical training programs.^23^ In Canada, the standard ITER assesses the seven CanMEDS roles identified by the Royal College of Physicians and Surgeons of Canada (2014) determining clinical competence for a physician. These seven roles are medical expert, scholar, communicator, collaborator, manager, professional and health advocate. Assessment of these various roles in the clinical setting is, however, complex and can pose many challenges, even when learners are regularly assessed.^5^ A survey of medical program directors in Canada found that they were relatively satisfied with their assessment of the *Medical Expert* role, but less so with assessment of the other CanMEDS roles, including communicator, collaborator, manager, professional and health advocate.^6^,^7^ Current approaches to validating assessment practices focus on the need for validation to be tailored to the specific context and to examine the assumptions underlining each aspect of an assessment structure.^11^ In the context that there are seven CanMEDS roles, insufficient work has been done to validate ITERs as a means of appropriately assessing each of these roles within the different educational contexts across medical education in Canada.

Direct observation of the resident in practice, on which the ITER is primarily based, has long been a part of medical education. As Fromme *et al*. (2009) points out, direct observation is “embedded in the medical education and apprenticeship process.”^8^ While the ITER plays a significant role in resident assessment, concerns have been raised about its accuracy and reliability.^3^,^12^ These concerns relate to a range of issues, including a lack of defined standards leading to inter-evaluator variation, fragmented observation of residents and a lack of timeliness in feedback delivery. A study of the use of the ITER for medical clerks in Western Canada identified issues around its validity, including a “halo effect,” in which raters simply selected the same category throughout the entire assessment.^14^ Perhaps related to this issue of inadequate assessments, few medical programs provide clinical faculty members with specific training in how to complete the ITER.^18^ There is also a significant discrepancy in how faculty and residents perceive the feedback given. In a study by Sender-Liberman et al., residents’ responses differed significantly from those of faculty on the frequency, timeliness, specific nature of feedback and overall effectiveness of the ITER on learning.^19^

As a result of the multiple challenges surrounding ITERs and their importance in medical evaluation, there has been a growing interest in examining faculty and resident attitudes toward the ITER process.^23^,^24^ The purpose of our study is to explore pediatric faculty and resident perspectives on the structure and process of the ITER, with the aim of improving the use of the ITER as an assessment tool. By eliciting these perspectives through separate focus groups, this study examines the attitudes of faculty on their approach and residents on their incorporation of the ITER within a pediatric residency program. Separating the focus groups also allows for the opportunity to compare the perspectives of residents and clinical faculty. To the best of our knowledge, our study is the first to focus on the ITER process in a pediatric residency training program and adds to the relatively small body of work on this topic done in Canada. In our discussions with other pediatric residency programs across the country, the ITER form and process used in the Memorial University of Newfoundland (MUN) pediatric residency program is similar to the process used in most other Canadian residency programs. In this way, we hope that the findings that we have found in our program will be of use to other residency programs across the country.

The study was conducted with residents and clinical faculty in the Division of Pediatrics at Memorial University of Newfoundland. This program offers a four-year residency program in pediatrics. All rotations use a standard ITER (Appendix 1), which is completed by the faculty member assigned supervisory responsibility for the rotation. The ITER form is sent directly to faculty via a computer-based One45 system, commonly used by many residency training programs.^16^ The ITER form directly asks the faculty member to indicate whether the residents performed “above expectations,” “at expectations” or “below expectations” on each of the seven CanMEDS roles and on the rotation overall using tick boxes. The supervising faculty member for the rotation is also encouraged to provide written comments related to each role. Finally, the ITER form asks the faculty member to indicate whether he or she met the resident to discuss their performance on the rotation prior to submitting the ITER. As part of the process, it is expected that faculty meet with residents to provide formative feedback mid-rotation and complete the ITER with the resident at the end of the rotation.

## Methods

All of the faculty and residents in the program were asked to participate in the project. A focus group methodology was selected to allow for a shared discussion of the issues arising for each group.^21^ Recruitment was conducted through a departmental e-mail sent to all clinical faculty and residents in the division of pediatrics, with people being asked to indicate their interest in participating in appropriate focus group. Each focus group was designed to have a minimum of 5 and a maximum of 9 people,^22^ with the plan being that there would be multiple focus groups if warranted by the number of participants. Based on the number of positive responses from potential participants, two separate focus groups were conducted in April 2012, within the MUN pediatric residency training program, the first with pediatric residents, followed by a second one with members of the clinical faculty. Eight of twenty-three (35%) invited residents attended (7 female, 1 male, N=8), representing a spectrum of training years (3 PGY-1; 3 PGY-2; 2 PGY-3). For the staff focus group, nine of forty (23%) invited attended (5 female, 4 male, N=9), which included the following specialties: cardiology, gastroenterology, infectious disease, developmental pediatrics, emergency medicine and general pediatrics. None of those who initially agreed to participate subsequently dropped out of the study. Except for one of the staff members who had received an ITER training session at the Royal College more than 10 years ago, no other staff member had received any formal training in ITER completion.

Consent forms and focus group discussion questions were sent to participants prior to the focus group, with written consent being obtained from all participants prior to the focus group sessions. All focus groups were facilitated by one of the researchers (RP), who was a resident in the program at the time and who had received graduate level training in qualitative research. Semi-structured focus group questions (Appendix 2) were developed by the authors, who include a faculty member with expertise in medical education (AD) and a PhD trained qualitative researcher (RC), based on the study aims and the literature review for the project, with the aim that they would direct the conversation to the key aspects of the ITER. Because the focus groups were all facilitated using a semi-structured interview guide, participants had the opportunity to ask for clarification of questions and to raise other issues that they thought were relevant to the project. Focus group sessions lasted approximately one hour and were all audio recorded and professionally transcribed. An assistant (DA) was present at each focus group to take written notes. Only the participants and the facilitators were present during the focus group sessions.

The data were analyzed using a thematic content analysis approach,^9^ with the aim of identifying the issues and suggested improvements each group had for the ITER. All of the transcripts and field notes were reviewed in their entirety by one of the authors (RP) and a research assistant (DA) before coding to ensure completeness and develop initial coding categories. One of the authors (RP) and a research assistant then both coded the data using NVivo 9 software,^15^ with both deductive and inductive coding being used.^4^ The coding of the first focus group transcript was reviewed by another author (RC), to validate the consistency of the coding. Key themes were then discussed and clarified by three of the authors. Ethics approval for the project was obtained from the *Newfoundland and Labrador Health Research Ethics Authority.*^10^

## Results

Both the pediatric faculty and residents felt that significant improvements can be made to optimize the use of ITERs, with both groups having numerous concerns and recommendations. We broadly categorized results under the themes of ITER format, understanding feedback, recording observations, verbal feedback, attitudes towards criticism, follow-up, and engagement and timeliness.

### ITER Format

Memorial’s pediatric program uses the same ITER form for each rotation. Residents were overall supportive of the basic design of the ITER, with its focus on the seven CanMEDS roles. While also supportive of the basic design, the clinical faculty requested that the form include more specific questions, particularly related to assessing the non-medical expert CanMEDS roles within clinical situations, with the aim of more clearly specifying the type of information that should be considered for each role. Some residents suggested that a rotation or year specific ITER may be a clearer way of setting expectations. Residents also felt that rotation specific ITERs would help with self-assessment, by focusing on a limited number of CanMEDS roles that are emphasized in one setting over another. Faculty also recognized the advantages of specific ITER assessments, including that they could help specify CanMEDS roles which should be emphasized in varying contexts. Another suggestion from a faculty member was for a group of similar rotations, e.g., sub-specialties rotations, to share a generic form, which would be a compromise between having a single form for the entire program and specific forms for each rotations.

### Understanding feedback

Given its role as an assessment instrument, it is not surprising that much of the discussion focused on feedback. A main component of the feedback that residents receive from the ITER is checkbox indications whether they performed at, above or below expectations. Residents unanimously agreed that there was a lack of understanding of what is meant by these grading scales and expressed a strong desire for a better definition of the grading scales on the ITER to reduce the variation in the criteria and standards that different clinical faculty use in assessing residents. In light of the problems in understanding the assessment categories, it is perhaps not surprising that all of the participants in the resident focus group felt that written comments were the most useful aspect of the ITER and should be mandatory. As one resident participant explained, “the written comments… are the most helpful because you get a sense of what the staff person was actually thinking as oppose (*sic*) to just the tick boxes...” Other residents went further, requesting that mandatory written comments should include discussion of both the strengths and areas of improvement for the resident based on the performance on the rotation.

Staff also expressed their confusion with the rating scales and that clearer definitions of the assessment categories on the ITER form were necessary. Part of the issue is that residents are expected to perform at a high level. One faculty participant recounted the instructions on an ITER from another Canadian pediatrics program, which said “excellence is expected and therefore average is excellent.” On the other end of the spectrum, another faculty participant said if “below expectations” is interpreted as a fail then “at expectations” could be seen as barely passing. While acknowledging that more written comments could be given, a few clinical faculty members identified concerns with the permanent nature of written comments and the potential impact they could have. One clinical faculty member said: “How much do you actually want to put on paper? Just having finished… reviewing fellowship applications, it’s sad to see how much weight is given to a slightly negative comment that might appear in their application …so writing something negative, I find to be very challenging.” Faculty also felt that the non-medical expert CanMEDS (i.e., the health advocate) roles are often difficult to assess in the clinical setting and that the program director and chief residents may be more appropriate to make more global assessments on these types of roles.

### Recording time and roles observed

Residents identified that better contextualization is important for setting expectations, self-assessment and external assessments of ITERs (e.g., by program or fellowship directors). One particular issue of concern was identifying how much time was spent with the clinical faculty member who is responsible for completing the ITER and the type of exposure that they had to the resident, which is important for providing the appropriate context and fair interpretation. Residents noted that clinical faculty members sometimes are not actually exposed to the various skills they are responsible for assessing, yet may still indicate the resident’s performance on the ITER form. As one resident said, “a lot of times I feel that the non-applicable (*sic*) box should be ticked instead, because I know you haven’t seen me do anything about this [CanMEDS role] on this rotation, and they just go through and fill them out anyway.”

Faculty also universally agreed that it is important for someone assessing the ITER to know how long the faculty member completing the form actually spent with the resident during the rotation, as there can be a great deal of variation in the amount of time the faculty member responsible for evaluating the rotation actually works with the resident. One of the participants in the faculty focus groups suggested that the first question on the ITER should be “how much time did you spend with this individual [on this rotation]?” Faculty members did discuss the challenge of commenting on the various CanMEDS roles with very little exposure. Yet the clinical faculty members also expressed some uncertainty about when it is appropriate to indicate ‘not applicable’ for a certain CanMEDS role. As one participant in the faculty focus group said, “the biggest problem is that a lot of times, looking at it, I say, jeez, I saw them once or twice.” For some rotations, the faculty members suggested it may be appropriate for other clinical persons to be invited to contribute to the assessment, if they worked more closely with the resident.

### Verbal feedback

Both residents and clinical faculty cited the importance of including verbal feedback as part of the ITER process. Verbal feedback facilitates a deeper conversation into issues that may have arisen within the rotations. It can also allow for discussions of topics that the faculty member might not feel comfortable putting in writing and making part of the resident’s permanent file. Residents said they greatly appreciated the effort made by faculty to discuss their performance because it assists with their own self-assessment process. As one resident said, “if someone is going to sit down and truly put the effort in and they give you verbal feedback, then you understand where they are coming from. The more detailed, the better it is for our own self-assessment.” All faculty members agreed that the preferred mode of delivering constructive criticism is “face-to-face.” Faculty members also emphasized that importance of verbal feedback when there are concerns or issues that need to be addressed with the resident. As one faculty member said, “the ITER is one thing, but I think you have to talk to the person and I think it’s when something appears in the ITER and no one has ever talked to the trainee, that people become disgruntled and [complain] that’s not fair.”

### Attitudes towards criticism

While faculty members recognize the importance verbal feedback, they acknowledge the challenge of having ‘difficult conversations’ with residents and that there is sometimes a culture within medicine where the critical discussions with residents are often avoided. Faculty members emphasized that residents should not get so hung up over negative feedback recognizing that humility is an important trait in medicine and it is an expected part of medical training to identify areas where performance needs to be improved. Faculty also admitted that the extent of the relationship that they have with a resident is a factor when considering giving negative criticism. As one faculty member said “one interaction with them [a resident] and you begin to wonder if it is a bad day, but if you have lot of experience with them, you see a trend and you feel more comfortable to say what you have seen is accurate.”

### Follow up

Residents felt that follow up from faculty plays an important part role in the ITER process. Opportunities to discuss performance half way through the rotation and setting out a learning plan are one option for helping to ensure progress. They noted that a plan or system for follow up should improvements be required needs to be in place, with some mechanism for reevaluation. While faculty members did not discuss remediation or having formal plans in place, one staff member mentioned the importance of letting residents know that there are people that can be approached to talk to if they receive a negative assessment. Another member then followed up by adding that staff should be occasionally reviewing a resident’s progress if it is found to be deficient.

### Engagement and timeliness

Faculty engagement and timeliness of feedback were most frequently cited as the most critical aspects for the ITER process that required improvement. Residents noted that late feedback has very little impact on their development. Most residents said that they had received either the infrequent written feedback, lack of verbal feedback, virtually no observation of skills, infrequent knowledge assessment and month long delays in receiving the ITER. As one resident reported, “I got an ITER from a person I barely worked with and I got it 4 months late.” There was also a strong request on the part of the residents for more opportunities to have their knowledge assessed, since they feel this was not happening frequently within specific rotations. While faculty members all acknowledge that they can be more involved with the process by providing more feedback of higher quality, they wish to do so in way that is sustainable and not completely monopolizing of their time. Overall, the residents felt that faculty members need to be more responsible for the entire ITER process and should be held more accountable for insufficient or delayed assessments, since teaching and assessment is part of their faculty duties.

We summarize the main recommendations for improving the ITER where there was agreement between the resident and clinical faculty focus groups in Table 1; and recommendations only made in one focus group in Table 2.

## Discussion

Our study examined faculty and resident perspectives on how to improve ITER evaluations in a pediatric program. While recognizing the potential role of the ITER, residents were frustrated by the limited exposure which clinical evaluators sometimes have of the residents they are assessing, receiving feedback well after the completion of a rotation, and the lack of acknowledgement of good resident performance. The recommendations discussed include the need to clarify the grading standards for the ITER; the expanded use of rotation specific assessments; requiring both written and verbal feedback; that negative feedback should include constructive criticism, given face-to-face in a timely manner; describing the exposure the evaluator had of the resident’s performance; and making clinical faculty more accountable for providing sufficient and timely assessments.

Though residents and faculty members shared a number of common concerns and suggestions for improving the ITER there were still areas of disagreements. Perhaps the biggest divide between faculty and residents is the quality of feedback sought by residents through the ITER process and the faculty member’s ability to do so in a timely way. Watling et al. (2008) found that engagement is central to the resident experience and the value residents placed on ITERs.^24^ From the evaluator perspective, engagement is demonstrated by grounding opinions in repeated, direct observation and by offering timely, specific and personalized feedback.^24^ When this preceptor engagement is absent, the resulting feedback is perceived by residents as meaningless and is unlikely to motivate behavior change.^24^ However, assessment is a two way street, therefore resident engagement (seeking as well as listening and insightfully responding to feedback) must also be present for the ITER to help bring about behaviour change.^23^

Paradoxically, while residents report that they would like more constructive criticism, they seem to have difficulty accepting it.^24^ We believe that residency training is a challenging time where resident self-esteem and confidence can be fragile and is built up through long hours and perseverance. Suddenly, with feedback and criticism, their internal experience becomes externalized and exposed. Why is this an issue? There exists a “competitive, high expectations” culture in medicine that demands high standards of physician competence. In this culture one’s weaknesses or mistakes can be criticized harshly. Our hypothesis is that in the run of busy day, staff may forget what it was like to be a resident trainee. Preceptors would do well to recognize this culture and time period of training from the residents perspective in order to make the process a more constructive one. We also agree with Watling et al. (2010) who concluded that the individuals who do best with constructive criticism are those that refrain from blaming others and try to learn as much as possible from the information and the experience.

Both faculty and residents may benefit from considering each other’s perspectives and explore sources of mutual misunderstandings based on common human cognitive biases such as the Fundamental Attribution Error and the Actor-Observer asymmetry. The Fundamental Attribution Error is one in which an individual attributes their own success to their skills and abilities and their failures to external circumstances while attributing the successes of others to external factors and their failures to character or internal shortcomings.^25^ The Actor-Observer asymmetry occurs when the observer purports to know the intentions of the actor and makes inferences or judgments about the actor.^25^ Here are a few examples to illustrate the above concepts. First, a resident explains their delay in answering pages on call to having multiple tasks at one time while the staff or preceptor would attribute their behavior to the resident’s inherent character flaws of being lazy, slow or forgetful (Fundamental Attribution error). Second, over a period of time, a resident cannot remember answers to questions when being tested by the staff because they have external stressors, post call fatigue or needed more time to think where the attending may attribute the lack of appropriate answers to having poor knowledge or being less competent (fundamental attribution error). In a final example, the resident shrugs her shoulders when responding to a concern made by a patient. This is interpreted in a particular (negative) way by the attending that was not intended by the resident. As a result the staff, failing to check on his/her assumptions, has a lower opinion of the character and skills of the resident (Actor-Observer asymmetry). The point in considering these common biases is to reduce their occurrence and to encourage communication between trainee (actor) and staff (observer) to better understand and therefore minimize their effects.

Perhaps the clinical or “hands on” assessment and educational system needs to be restructured given that physicians often do not have enough time to thoroughly observe, correct, assist and speak to trainees to provide the meaningful feedback that would more effectively contribute to resident learning and progress. Accreditation of all undergraduate and postgraduate training programs in North America requires that the clinical skills of trainees be assessed by direct faculty observation^26^. If this is to remain the standard for accreditation, based on the findings of our study and previous literature regarding faculty engagement, significant changes should be considered. One option is to have residents play a more proactive role in the assessment process by approaching staff to increase the number of opportunities for verbal feedback, putting less emphasis on written feedback. Future research can also look further into how residents self-assess and the process of introspection that residents go through when receiving feedback. Finally, with recommendations from the Accreditation Council for Graduate Medical Education (ACGME) to implement 360 degree multi source as a valid assessment tool in post graduate training, further research will be needed to determine how the ITERs will fit into this process and the reliability challenges that exist when incorporating a larger group of individuals into the assessment forum 1,2.

The findings of this study need to be considered in terms of the proposed revisions to the Royal College’s 2015 CanMEDS roles. The new approach will include “the addition of milestones, which are being created for each role included in the CanMEDS Framework,” which will continue across a physician’s career. While the new approach has not yet been fully articulated, the aim of clarifying the roles and expressing the aims of each role in simpler, more direct, language should help addressed some of the concerns expressed by faculty in project about the confusions regarding the expectations for non-medical CanMEDS roles. The incorporation of a milestones approach should also allow for faculty to clarify the expectations at different years of residency.

One limitation in our study is that it was conducted within a single pediatric program. However, given that the ITER is used in over 90% of post graduate training programs and that the ITER used in the pediatric program studied includes elements assessed across residency programs, our findings likely have some applicability to other Canadian residency programs 26. It should be noted that the researcher who conducted the focus groups (RP) was a resident in the program at the time that the work was conducted. Both focus groups were made aware that information disclosed would remain completely confidential which allowed both sides to be candid in their comments. However, in the resident focus group, the resident conducting the focus group would have been regarded as a peer, while in the staff focus group the resident may have represented a source by which information could be leaked out to other trainees and thus comments may have been restricted. While all staff clinical faculty and residents in the program were invited to participate, the response rate was relatively low at 23% and 35% respectively. It is also unlikely that two one-hour focus group sessions allowed for data saturation of all of the relevant issues. Finally, a high proportion of the participants in the resident focus group were female. While the percentage (88%) is in keeping with the makeup of the pediatric residency program studied (21 females and 3 males), this distribution could possibly bias comments and suggestions made during the focus groups compared to programs with a more gender balanced program. However a predominance of females is a common situation for most pediatric programs across Canada. In 2013, the Canadian Medical Association published a pediatrics profile that showed that in the 2012/2013, of the total 156 new first year residents, 130 (83%) were female 27. The Division of Pediatrics has already moved to address some of the issues raised during our project. When asked about ITER training, only one staff member in the focus group had ever received any ITER training, which was done years ago by the Royal College. None of the staff had received any formal training on how to fill out the new CanMEDS-based ITER that is currently in use. This lack of consistent training likely adds to the lack of clarity in the grading criteria used. In reaction to this perceived need, Memorial’s Division of Pediatrics recently held a training workshop with clinical faculty on trainee assessment and the ITER process. Future research into the impact of an ITER training program on resident assessment would be worth examining. The program has also moved to improve the timeliness of ITERs, by sending more reminders and requiring a meeting with the program chair to explain long delays in the completion of ITERs by faculty, which appears to have reduced the time that ITERs are outstanding. Other actions that could be taken are to further specify the expectations around the fulfillment of the non-medical CanMEDS and requiring more oral and written feedback throughout rotations. Our results also likely raise issues for the use of ITERs in clerkship assessments. Future research could include a survey of residents and clinical faculty in other programs and schools across Canada to determine the extent to which they share the same ideas about the use of and improvements in ITERs especially considering the upcoming revisions to the CanMEDS roles.

## Figures and Tables

**Table 1:** Recommendations mutually agreed upon by faculty and residents

The need for a clearer understanding of the grading standards of the ITER The need for rotation specific ITERs to help focus on specific CanMEDS objectives ITERs should record the length of time spent with trainee and the level of interaction Each ITER should be accompanied by both written and verbal feedback The faculty who spends the most time with a resident on their rotation should be responsible for filling out the ITER Constructive criticism and negative feedback should be timely and provided face to face Residents should be more involved in the ITER process and in setting rotation learning objectives

**Table 2:** Recommendations only made in one focus group

Recommendations only by faculty	Recommendations only by residents

Involvement of other health care professionals is required Program Director and chief residents may be more appropriate to make global assessments for non-medical expert CanMEDS roles Residents need to be more open when receiving constructive criticism Residents should be aware that ‘meeting expectations’ means performance is at par and that most individuals will fall into this category	Narrative comments should be mandatory Timeliness of feedback is critical Faculty must observe encounters and skills to provide meaningful feedback Formal mechanisms should be in place so that progress can be tracked Faculty ITER training is necessary Clinical faculty should be more accountable for providing sufficient and timely evaluations

## Appendix I: Focus Group Questions

### Questions for Faculty Focus Group

1. How important do you feel the ITER is in overall resident evaluation during residency?
  - Do you feel that the ITER is meaningful and provides a fair assessment for a resident’s performance?
2. Have you ever received any training in how to conduct and assess an ITER?
3. What are the strengths and weaknesses of the ITER as an evaluation tool?
4. What suggestions would you make for improvement of this evaluation tool?
5. What if any are the biggest barriers in the process of filling out the ITER completely for yourself?
6. Which CanMED roles on the ITER are most difficult to comment on and why? (medical expert, scholar, communicator, collaborator, manager, professional and health advocate)
7. What is your preferred method (ie written, verbal) of delivering constructive criticism for resident performance?
8. What is your experience with receptivity of constructive criticism from residents? Are there often disagreements?
9. Do the classification scheme for grading typically reflect your thoughts of resident performance? Should there be more defined scales with more intervals?
10. How do you think that resident performance should be assessed?

### Questions for Resident Focus Group

1. How important do you feel the ITER is in your training to becoming a competent pediatrician?
2. What aspect of the ITER is most helpful?
3. What factors do you consider before placing weight on the comments?
4. Is self-assessment a big part of your feedback and progress?
  - Does the ITER feedback typically match your own self-assessment?
5. What are the strengths and weaknesses of the ITER as an evaluation tool?
6. What suggestions would you make for improvement of this evaluation tool?
7. Do you seek verbal feedback in addition to your ITER?
8. What is your preferred mode of receiving constructive criticism?
9. Does the classification scheme reflect how you feel you are doing or are more specific intervals required to describe performance (ie faculty evaluation is more detailed and is based on a 5 pt scale)
10. Do you have any suggestions for faculty in helping them fill out the ITER?
